# A comparative study on percutaneous vertebroplasty for osteoporotic vertebral compression fractures under day surgery and inpatient management models

**DOI:** 10.3389/fsurg.2026.1880663

**Published:** 2026-07-14

**Authors:** Yufei Yuan, Ye Tian, Pengfei Shen, Jun Miao

**Affiliations:** 1 Clinical School/College of Orthopedics, Tianjin Medical University, Tianjin, China; 2 Biomedical Engineering, Academic Division of Medicine, Tianjin University, Tianjin, China; 3 Department of Spine Surgery, Tianjin Hospital, Tianjin University, Tianjin, China

**Keywords:** day surgery, inpatient management, osteoporotic vertebral compression fracture, Oswestry Disability Index, percutaneous vertebroplasty

## Abstract

**Objective:**

To compare the clinical efficacy, safety and health economic differences of percutaneous vertebroplasty for osteoporotic vertebral compression fractures (OVCF) under the day surgery and conventional inpatient management modes.

**Methods:**

A retrospective cohort study was conducted. A total of 168 OVCF patients who underwent percutaneous vertebroplasty from March 2023 to December 2024 were included, with 86 cases in the day surgery group, who completed admission, surgery, postoperative observation and discharge within one working day, and 82 cases in the inpatient group, who stayed overnight after surgery or had a total hospital stay of more than 24 h. The grouping was based on: patients who met the hospital's day surgery admission criteria and had perfect home care conditions after receiving standardized outpatient evaluation, anesthesia evaluation and perioperative risk stratification were included in the day surgery pathway; those who adopted the conventional inpatient process due to comorbidities requiring extended observation, long preoperative evaluation time, insufficient home care or patient's wishes were included in the inpatient group. The visual analogue scale (VAS) score, Oswestry disability index (ODI), analgesic medication use score and mobility score were compared between the two groups before surgery, 1 week after surgery and at the last follow-up. Meanwhile, the direct medical costs, patient satisfaction, surgery-related indicators and perioperative complications were compared between the two groups.

**Results:**

There were no statistically significant differences in baseline data between the two groups (all *P* > 0.05). The VAS score, ODI, analgesic medication use score and mobility score of the day surgery group at 1 week after surgery and at the last follow-up were significantly improved compared with those before surgery (all *P* < 0.01). There were no statistically significant differences in VAS score, ODI, analgesic medication use score and mobility score between the two groups at 1 week after surgery and at the last follow-up (all *P* > 0.05). The total hospital stay of the day surgery group was shorter than that of the inpatient group, the direct medical cost was lower than that of the inpatient group, and the patient satisfaction was higher than that of the inpatient group, with statistically significant differences (all *P* < 0.01). There were no statistically significant differences in intraoperative blood loss, bone cement injection volume and postoperative complication rate between the two groups (all *P* > 0.05).

**Conclusion:**

For strictly screened OVCF patients, percutaneous vertebroplasty under the day surgery mode can achieve short-term and early medium-term efficacy equivalent to that of inpatient management, without increasing the risk of perioperative complications, and can significantly shorten the hospital stay, reduce direct medical costs and improve patient satisfaction.

## 1 Introduction

Osteoporotic vertebral compression fracture (OVCF) is one of the most common brittle fractures in the elderly, which can cause persistent low back pain, limited spinal mobility, dysfunction and decreased quality of life (1). With the deepening of aging, the demand for diagnosis and treatment related to OVCF continues to increase (2). Percutaneous vertebroplasty has become an important treatment option for patients with ineffective conservative treatment due to its advantages of minimal trauma, rapid onset of effect, and promotion of early activity (1). In recent years, the day surgery model has developed rapidly in surgical practice, and its core lies in achieving efficient diagnosis and treatment on the premise of safety through pre-operative assessment advancement, standardized peri-operative procedures and continuous post-discharge management (3). However, OVCF patients are usually older, often complicated with cardiovascular and cerebrovascular diseases, diabetes and multiple risks related to osteoporosis, and whether day surgery can reflect economic advantages while ensuring efficacy and safety remains to be further evaluated (4). Therefore, this study retrospectively compared the clinical outcomes, safety and cost differences of percutaneous vertebroplasty for OVCF under day surgery and inpatient management models.

## 2 Subjects and methods

A retrospective cohort study design was adopted. Patients with OVCF who underwent percutaneous vertebroplasty in the Department of Orthopedics of Tianjin Hospital from March 2023 to December 2024 were selected. Inclusion criteria: (1) Age ≥60 years; (2) Clear thoracolumbar pain symptoms and corresponding signs of tenderness and percussion pain; (3) Imaging examinations indicate fresh vertebral compression fractures, including bone marrow edema signals shown by magnetic resonance imaging or increased metabolism of the responsible vertebral body suggested by radionuclide imaging; (4) Received simple percutaneous vertebroplasty; (5) Complete follow-up data, with follow-up time ≥12 months. Exclusion criteria: (1) Pathological fractures caused by tumors, infections, tuberculosis, etc.; (2) Combined with obvious neurological dysfunction requiring open decompression and fixation; (3) Those with uncorrected coagulation dysfunction; (4) Multiple severe traumas or those who underwent other spinal surgeries at the same stage; (5) Those with missing clinical data.

A total of 168 patients were enrolled, including 86 cases in the day surgery group and 82 cases in the inpatient group. In the day surgery group, there were 32 males and 54 females, with an age of (73.4 ± 6.8) years; in the inpatient group, there were 29 males and 53 females, with an age of (74.1 ± 7.2) years. There were no statistically significant differences in gender, age, body mass index, number of fracture segments, composition of underlying diseases and ASA classification between the two groups (all *P* > 0.05), as shown in Table 1.

**Table 1:** Comparison of baseline characteristics between the two groups of patients.

Group	Day surgery group (*n* = 86)	Inpatient group (*n* = 82)	Statistical value	*P* value
Male/Female (*n*)	32/54	29/53	*χ*² = =0.087	>0.05
Age ($\bar{x}\pm s$, years)	73.4 ± 6.8	74.1 ± 7.2	*t* = 0.649	>0.05
Body Mass Index ($\bar{x}\pm s$, kg/m^2^)	23.1 ± 3.2	22.8 ± 3.5	*t* = 0.580	>0.05
Number of fracture segments per case ($\bar{x}\pm s$, *n*)	1.16 ± 0.37	1.13 ± 0.35	*t* = 0.541	>0.05
Hypertension [cases (%)]	46 **(**53.5**)**	42 **(**51.2**)**	χ² = 0.090	>0.05
Diabetes [cases (%)]	19 **(**22.1**)**	17 **(**20.7**)**	χ² = 0.046	>0.05
Coronary heart disease [cases (%)]	15 **(**17.4**)**	16 **(**19.5**)**	χ² = 0.119	>0.05
ASA classification I/II/III/IV (*n*)	12/29/39/6	10/27/38/7	χ² = 0.405	>0.05
Last follow-up time (month)	12.8 ± 5.6	13.2 ± 6.2	*t* = 0.439	>0.05

ASA stands for the American Society of Anesthesiologists grading.

This study was approved by the Ethics Committee of Tianjin Hospital (Batch No.: 2025-179), and all patients signed informed consent forms.

### 21 Grouping principle

This study adopted a non-random grouping method based on real clinical decision-making pathways. All patients first completed a preliminary diagnosis in the outpatient or emergency department, and after being confirmed by spinal surgeons to meet the indications for percutaneous vertebroplasty, they uniformly entered the preoperative assessment process. Afterwards, the departments of orthopedics, anesthesiology and the day surgery management unit jointly completed the perioperative risk stratification, and grouped them according to the following principles.

Inclusion in the day surgery group requires simultaneous satisfaction of the following conditions: (1) The responsible vertebral body is clearly defined, and standardized percutaneous vertebroplasty is to be performed; (2) Preoperative laboratory tests, electrocardiogram and chest imaging show no obvious abnormalities, or there are chronic underlying diseases but they are well-controlled without the need for further in-hospital adjustment; (3) The majority of patients have an ASA physical status classification of grade Ⅰ to Ⅲ, and a small number of grade Ⅳ patients who can safely undergo surgery under monitored local anesthesia after multi-disciplinary assessment can also be included; (4) The patient's expected postoperative pain is controllable, without the need for long-term intravenous analgesia or continuous monitoring; (5) Have conditions including family accompanying care, 24-hour emergency hospital return access, and accessibility for postoperative telephone follow-up; (6) The patient and their family understand and agree to the day surgery procedure (5–7).

The main reasons for being included in the inpatient group include: (1) Comorbidities that require further preoperative optimization, such as fluctuations in blood pressure or blood glucose, adjustment of anticoagulant drugs, and arrhythmia to be observed, etc.; (2) The anesthesiology department assesses that prolonged postoperative observation is needed; (3) The patient is elderly, has a high degree of frailty or lives alone, and has insufficient off-hospital care conditions; (4) The patient or their family actively choose routine inpatient management for safety considerations (5, 6).

To reduce selection bias, this study compared the baseline characteristics of the two groups of cases, including age, gender, number of fracture segments, composition of underlying diseases, and ASA classification, during inclusion and analysis. The results showed that the two groups were basically balanced, as shown in Table 1.

### 22 Surgical treatment

Both groups underwent standard percutaneous vertebroplasty (5, 8, 9). Patients were placed in the prone position, and under the guidance of C-arm x-ray fluoroscopy, puncture was performed via the unilateral or bilateral pedicle approach to the anterior-middle part of the responsible vertebral body. After establishing the working channel, polymethylmethacrylate bone cement was slowly injected under continuous fluoroscopic monitoring, and the injection was immediately stopped when the bone cement distribution was satisfactory or there was a trend of leakage. After the operation, the puncture needle was removed and local compression bandaging was performed. All surgeries were completed by the same medical team to reduce the impact of operator differences on outcomes.

### 2.3 Perioperative management

Both groups of patients received local infiltration anesthesia combined with anesthesia monitoring. Perioperative management specifically includes the following steps (5–7, 10).

1. Preoperative evaluation: The operating surgeon will complete medical history collection, physical examination and determination of the responsible vertebral body; the anesthesiologist will conduct airway, cardiopulmonary function and ASA classification evaluation; consult specialists such as cardiology and endocrinology departments when necessary.
2. Preoperative examinations: including blood routine, urine routine, coagulation function, liver and kidney function, electrolytes, blood glucose, infection screening, electrocardiogram and chest imaging examinations; for high-risk patients, add echocardiography or lower extremity venous ultrasound and other examinations.
3. Preoperative education: Explain to the patient and their family in detail the surgical procedure, anesthesia plan, risks such as bone cement leakage, postoperative functional exercise requirements, and anti-osteoporosis treatment requirements.
4. Intraoperative management: Continuously monitor blood pressure, heart rate, blood oxygen saturation and electrocardiogram changes; establish intravenous access; administer small doses of sedatives, analgesics or antihypertensive drugs based on changes in pain and vital signs.
5. Postoperative recovery: After the patient is returned to the recovery area or ward, conduct continuous observation for 2–6 h, focusing on assessing vital signs, bleeding at the puncture site, neurological function, pain relief degree and the ability to get out of bed and move.
6. Discharge instructions: including wound care, brace wearing, gradual weight-bearing activities, standardized use of analgesic drugs, anti-osteoporosis medication, prevention of refracture, warning of abnormal symptoms and indications for hospital revisit.
7. Follow-up management: Dedicated staff will make phone calls for follow-up within 24 h after surgery, and conduct phone or outpatient reexaminations at 1 week, 1 month, 3 months, 6 months and the final follow-up after surgery.

For the day surgery group, our hospital has established a standardized clinical pathway management model, which includes eight consecutive links: “outpatient screening—preoperative examination advancement—multidisciplinary assessment—surgery appointment—day surgery ward admission—short-term postoperative observation—discharge assessment—telephone follow-up”. This pathway is collaboratively implemented by the orthopedics department, anesthesiology department, day surgery center, nursing team and inpatient management department, and a unified admission criteria, risk assessment form, nursing checklist, discharge criteria and emergency inpatient transfer process have been formulated, so as to ensure the standardization and traceability of day surgery (5, 6).

Discharge criteria for day surgery are: (1) Stable vital signs for ≥2 h; (2) Clear consciousness and normal orientation; (3) Visual Analogue Scale (VAS) score ≤3 points; (4) Able to safely sit up, stand or walk short distances with assistance; (5) No progressive neurological abnormalities; (6) No active bleeding or significant exudation at the puncture site; (7) No complications requiring further inpatient management; (8) The patient has conditions for post-discharge care and unobstructed contact information. Patients who fail to meet any of the criteria will be transferred to an inpatient ward for further observation (5–7, 10).

### 2.4 Evaluation methods for efficacy and safety

1. Pain score: The VAS is used, with 0 points indicating no pain and 10 points indicating severe pain.
2. Oswestry Disability Index (ODI): Expressed as a percentage, a higher value indicates more severe low back disability.
3. Analgesic medication use score: 0 point means no analgesics used, 1 point means non-steroidal anti-inflammatory drugs, 2 points means intermittent oral weak opioids, 3 points means continuous oral opioids, 4 points means opioid injections required.
4. Mobility score: 1 point means walking independently, 2 points means walking with the help of braces or others, 3 points means wheeling a wheelchair, 4 points means staying in bed.
5. Health economics indicators: Direct medical expenses included hospitalization fees, surgical fees, anesthesia fees, material fees (including bone cement and puncture needles), medication fees, laboratory and examination fees, and nursing fees. Preoperative outpatient examination costs incurred prior to admission were also included in the total direct medical costs. All costs were calculated based on the gross charges billed to patients before any health insurance reimbursement, and the potential impact of varying health insurance reimbursement rates was not adjusted in the primary analysis, as this study focused on the overall resource consumption from the healthcare system perspective.
6. Patient Satisfaction: Adopt a 100-point satisfaction scale 1 week after discharge for evaluation, with higher scores indicating higher satisfaction. The scale included items regarding medical service, nursing care, pain management, and discharge process. A score≥80 was defined as satisfactory. This self-developed scale was reviewed by expert panels for content validity.
7. Safety indicators: including operation time, intraoperative blood loss, bone cement injection volume, bone cement leakage, hematoma or infection at the puncture site, nerve root irritation symptoms, refracture and serious adverse events.

### 2.5 Follow-up visit

A combination of outpatient reexamination and telephone follow-up was adopted. The follow-up time points were 1 week, 1 month, 6 months after surgery and the final follow-up. This study selected the data of preoperation, 1 week after surgery and the final follow-up for analysis. The average follow-up time was 13.1 months (12–22 months), among which the day surgery group was 12.8 months (11–21 months) and the inpatient group was 13.2 months (12–22 months).

## 3 Statistical methods

All statistical analyses were performed using SPSS 26.0 software. Continuous variables conforming to a normal distribution are presented as mean ± standard deviation, and comparisons between groups were made using independent samples *t*-test; comparisons at different time points within the same group were made using repeated measures analysis of variance. Non-normally distributed data are presented as median (interquartile range), and comparisons between groups were made using Mann–Whitney *U*-test. Categorical variables are presented as case number (percentage), and comparisons between groups were made using *χ*^2^-test or Fisher's exact test. To further control for potential selection bias and residual confounding, multivariate linear regression analyses were performed for continuous outcomes (e.g., direct medical costs, VAS score, ODI), and multivariate logistic regression analyses were conducted for categorical outcomes (e.g., complications), adjusting for age, gender, ASA classification, and comorbidities (hypertension, diabetes, coronary heart disease). All tests were two-sided, and *P* < 0.05 was considered statistically significant.

## 4 Results

### 4.1 Comparison of preoperative clinical data between the two groups of patients

A total of 196 fractured vertebrae were involved in 168 patients. There were 103 vertebrae in the day surgery group and 93 vertebrae in the inpatient group. There were no statistically significant differences between the two groups in terms of age, gender composition, body mass index, distribution of fracture segments, comorbid basic diseases and ASA classification (all *P* > 0.05), as shown in Table 1. In terms of bone cement leakage, the day surgery group had 6 cases (3 intradiscal, 2 paravertebral, 1 venous) and the inpatient group had 7 cases (4 intradiscal, 2 paravertebral, 1 epidural).

### 4.2 Comparison of postoperative efficacy, costs and satisfaction between the two groups of patients

**Figure 1:**
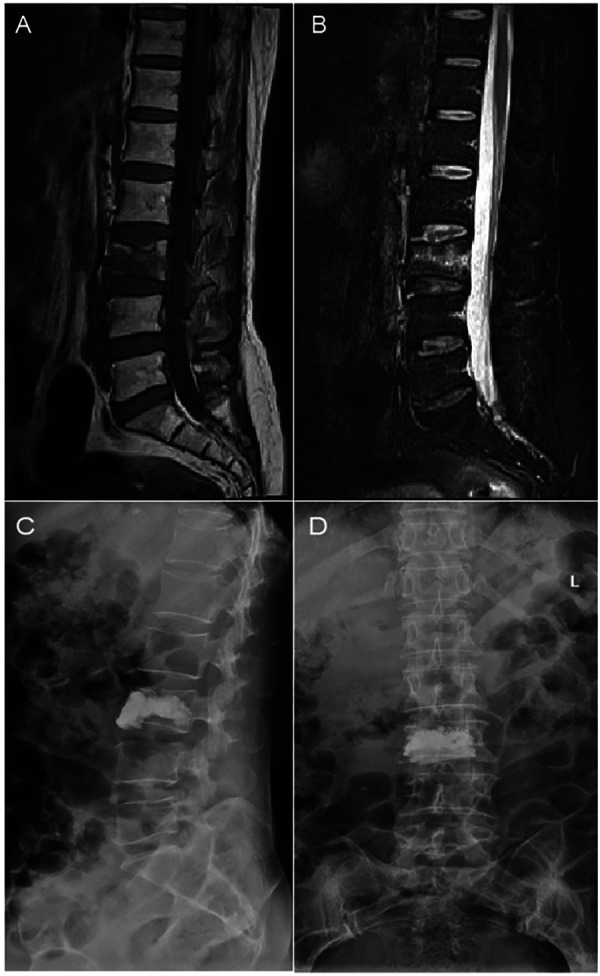
A 67-year-old female with OVCF in day surgery group. **(A,B)** MRI revealing L3 fracture before operation; **(C,D)** x-ray showing L3 with bone cement after PVP.

**Figure 2:**
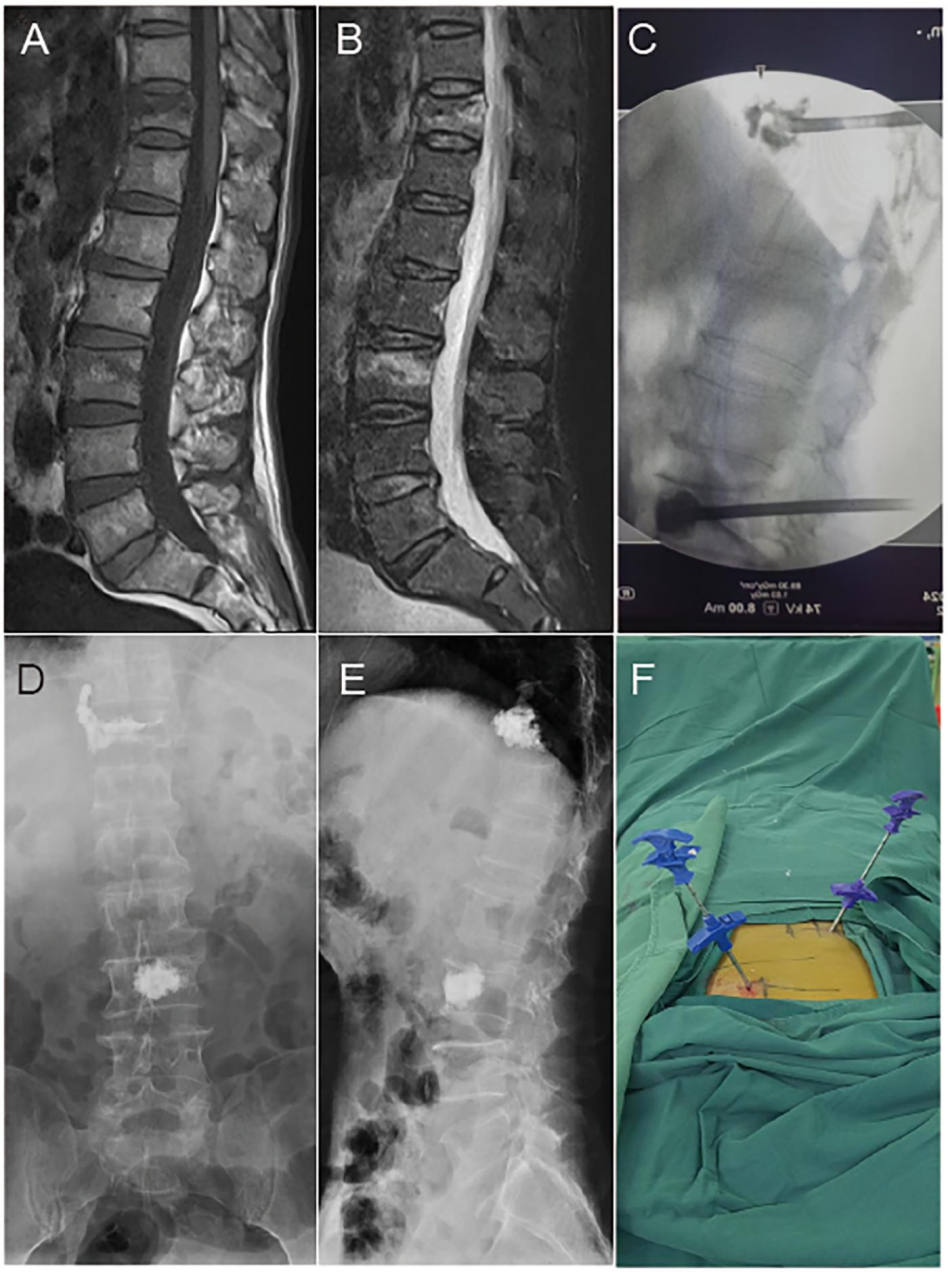
A 72-year-old female with OVCF in day surgery group. **(A,B)** Preoperative MRI reveals fractures at T11 and L3; **(C)** Intraoperative C-arm fluoroscopy image showing the procedure. **(D,E)** Postoperative x-ray showing T11 and L3 with bone cement after PVP. **(F)** Intraoperative photograph showing the bilateral puncture technique.

One week after surgery, the VAS scores, ODI scores, analgesic medication use scores and mobility scores of patients in the day surgery group were significantly improved compared with those before surgery, and the improvement persisted at the last follow-up, with statistically significant differences (all *P* < 0.01). The inpatient group showed the same changing trend. Comparison between the two groups showed that there were no statistically significant differences in VAS scores, ODI scores, analgesic medication use scores and mobility scores at 1 week after surgery and at the last follow-up (all *P* > 0.05). After adjusting for potential confounders including age, gender, ASA classification, and comorbidities, multivariate linear regression analysis confirmed that the day surgery group still had significantly lower direct medical costs (*β* = −2,911, 95% CI: −3,678 to −2,144, *P* < 0.001) and higher patient satisfaction (*β* = 3.6, 95% CI: 2.1 to 5.1, *P* < 0.001) compared to the inpatient group. No significant differences were found in VAS score, ODI, or complication rates between the two groups (all *P* > 0.05). The total length of hospital stay in the day surgery group was significantly shorter than that in the inpatient group, the direct medical costs were lower than those in the inpatient group, and the patient satisfaction was higher than that in the inpatient group, with statistically significant differences (all *P* < 0.01), as shown in Table 2. Further breakdown of the direct medical costs showed that the day surgery group had significantly lower expenses in hospital bed and nursing fees (*P* < 0.01) and medication fees (*P* < 0.01) compared to the inpatient group, while there were no significant differences in surgical fees, anesthesia fees, and material fees between the two groups (all *P* > 0.05). Typical images are shown in Figures 1, 2.

**Table 2:** Comparison of efficacy indicators between the two groups before and after surgery and at the last follow-up ($\bar{x}\pm s$).

Group	Day surgery group	Inpatient group	*t*-value	*P*-value
Number of cases	86	82	-	>0.05
VAS score before surgery	7.4 ± 1.0	7.2 ± 1.1	1.225	>0.05
VAS score 1 week after surgery	2.1 ± 1.1	2.3 ± 1.3	1.081	>0.05
VAS score at the last follow-up	1.5 ± 0.8	1.6 ± 0.9	0.760	>0.05
ODI before surgery	68.2 ± 8.5	67.5 ± 8.9	0.527	>0.05
ODI 1 week after surgery	28.6 ± 6.9	30.1 ± 7.2	1.380	>0.05
ODI at the last follow-up	19.8 ± 5.7	20.5 ± 6.0	0.782	>0.05
Analgesic score before surgery	2.5 ± 0.8	2.4 ± 0.9	0.758	>0.05
Analgesic score 1 week after surgery	1.2 ± 0.5	1.3 ± 0.6	1.157	>0.05
Analgesic score at the last follow-up	0.8 ± 0.4	0.9 ± 0.5	1.401	>0.05
Mobility score before surgery	2.8 ± 0.6	2.7 ± 0.7	1.012	>0.05
Mobility score 1 week after surgery	1.4 ± 0.6	1.5 ± 0.7	0.989	>0.05
Mobility score at the last follow-up	1.2 ± 0.5	1.3 ± 0.6	1.176	>0.05
Direct medical cost (Chinese Yuan, ¥, x ± s)	15,632 ± 2,158	18,543 ± 2,937	7.345	<0.01
Patient satisfaction	94.2 ± 4.8	90.6 ± 5.3	4.630	<0.01

In the day surgery group, there were statistically significant differences in all indicators between 1 week after surgery and at the last follow-up compared with before surgery (all *P* < 0.01); The comparison results within the inpatient group are the same.

### 4.3 Comparison of perioperative indicators and complications between the Two groups of patients

The operation time of the day surgery group was (34.5 ± 8.6) min, and that of the inpatient group was (36.1 ± 9.2) min; the intraoperative blood loss was (7.8 ± 3.4) mL and (8.2 ± 3.7) mL respectively; the bone cement injection volume was (4.6 ± 1.1) mL and (4.8 ± 1.2) mL respectively, with no statistically significant differences (all *P* > 0.05). In terms of complications, the day surgery group had 6 cases of bone cement leakage, 1 case of puncture site hematoma or infection, 2 cases of transient nerve root irritation symptoms, and 5 cases of refracture during follow-up; the inpatient group had 7, 2, 1, and 6 cases respectively. There was no statistically significant difference in the total incidence of complications between the two groups (*P* > 0.05). No pulmonary embolism, spinal cord injury, deep infection or perioperative death occurred in either group, see Table 3.

**Table 3:** Comparison of perioperative indicators and complications between the two groups of patients.

Group	Day surgery group (*n* = 86)	Inpatient group (*n* = 82)	Statistical value	*P*-value
Operation duration ($\bar{x}\pm s$, min)	34.5 ± 8.6	36.1 ± 9.2	*t* = 1.165	>0.05
Intraoperative blood loss ($\bar{x}\pm s$, mL)	7.8 ± 3.4	8.2 ± 3.7	*t* = 0.731	>0.05
Bone cement injection volume ($\bar{x}\pm s$, mL)	4.6 ± 1.1	4.8 ± 1.2	*t* = 1.128	>0.05
Total hospitalization time ($\bar{x}\pm s$)	9.6 ± 1.8 h	3.4 ± 1.1 d	*t* = 27.684	<0.01
Bone cement leakage [cases (%)]	6 **(**7.0**)**	7 **(**8.5**)**	χ^2^ = 0.138	>0.05
Hematoma or infection at puncture site [cases (%)]	1 **(**1.2**)**	2 **(**2.4**)**	Fisher	>0.05
Symptoms of nerve root irritation [cases (%)]	2 **(**2.3**)**	1 **(**1.2**)**	Fisher	>0.05
Refracture [cases (%)]	5 **(**5.8**)**	6 **(**7.3**)**	χ^2^ = 0.154	>0.05
Severe complications^a^ [cases (%)]	0 **(**0**)**	0 **(**0**)**	—	—

a Severe complications include pulmonary embolism, spinal cord injury, deep infection, and perioperative mortality.

## 5 Discussion

The results of this study show that after strict preoperative screening, patients with OVCF who undergo percutaneous vertebroplasty under the daytime surgery model have similar postoperative pain relief, recovery of lumbar and back function, reduced demand for analgesic drugs, and improved mobility to those managed under the inpatient hospitalization model, with no increase in perioperative complications. This suggests that for eligible patients, daytime surgery is not only feasible but also optimizes resource allocation while ensuring medical quality.

Percutaneous vertebroplasty itself has the technical basis for performing day surgery (5, 6). This procedure features a small incision, short operation time, and low blood loss. Most cases can be completed under local anesthesia combined with monitoring, with rapid postoperative recovery, which can meet the management requirements of “short-term observation and rapid discharge” (8, 9, 11, 12) At the same time, most OVCF patients are elderly populations. Long-term bed rest after fracture can increase the risks of pulmonary infection, constipation, venous thrombosis and disability. If surgery can be completed as soon as possible on the basis of standardized evaluation and patients can return to the home environment as soon as possible, it is theoretically helpful to reduce non-surgery-related hospitalization risks (5, 13). Consistent with this, the length of hospital stay in the day surgery group of this study was significantly shortened, and the main outcomes such as pain, ODI and mobility were not inferior to those of the inpatient group.

This study also shows that the direct medical costs of the day surgery group are lower than those of the inpatient group, and the patient satisfaction is higher than that of the inpatient group. It should be noted that the direct medical costs in this study were calculated based on gross charges prior to health insurance reimbursement. Different health insurance reimbursement policies and rates across regions may affect the actual out-of-pocket expenses for patients, which was a limitation of the current economic evaluation. Additionally, the inclusion of preoperative outpatient examination costs in our calculation reflects the true total medical resource consumption of the two pathways. The economic advantage of the day surgery model primarily stems from the reduction in hospital stay and the standardized clinical pathway, which significantly decreases bed and nursing costs. The possible reasons are as follows: first, the shortened hospital stay reduces the expenses for hospital beds, nursing care, and some examination and drug-related expenditures; second, the standardized pathway reduces the preoperative waiting time and process redundancy; third, patients complete diagnosis and treatment in a short period of time, which reduces the costs of repeated transfers, waiting and accompanying care, and brings a better subjective medical experience. It should be pointed out that the improvement of satisfaction does not mean that all patients are suitable for day surgery management, and safety should always be given top priority. Especially for the elderly, frail patients, those with multiple comorbidities and those with insufficient family support, flexible space for transferring to inpatient observation should be reserved.

The key to the successful implementation of the daytime surgery model does not lie in simply shortening the length of hospital stay, but in establishing a complete, standardized and enforceable clinical pathway (5, 6). The standardized pathway adopted in this study moves the preoperative assessment forward to the outpatient department, where the orthopedics department, anesthesiology department, daytime surgery center and nursing team jointly complete the admission check, and improve the quality of continuous care through unified discharge standards and postoperative follow-up processes. For percutaneous vertebroplasty, local anesthesia combined with monitoring also has obvious advantages: it can reduce the impact of general anesthesia on the cardiopulmonary function of elderly patients, while retaining the patient's response during the operation, which is conducive to timely identification of abnormalities related to nerve stimulation, thereby improving operational safety. Furthermore, it is worth noting that some patients in the inpatient group had a relatively short stay (e.g., around 28 h), which slightly overlaps with the day surgery threshold. This minimal difference in total hospital stay may dilute the observed differences in clinical and economic outcomes, suggesting that the actual advantages of the day surgery model could be more pronounced than reported.

In this study, the incidences of bone cement leakage, nerve root irritation symptoms, and refracture were similar between the two groups, and no severe disabling complications occurred. This suggests that under the guarantee of a mature team and standardized procedures, daytime surgery does not increase additional risks. Although leakage was reported as a binary event in this study, different types of leakage carry distinct clinical implications. Most leakages in our cohorts were intradiscal or paravertebral, which are often asymptomatic, whereas epidural or venous leakages pose higher neurological or pulmonary risks. Intraoperative control of bone cement viscosity, injection speed, and real-time fluoroscopic monitoring can greatly reduce its clinical impact (14, 15). The occurrence of refracture is related to the underlying osteoporosis, the severity of the primary fracture, and the compliance with anti-osteoporosis treatment, so long-term postoperative bone metabolism management is also equally important (16, 17).

This study still has several limitations. First, this is a single-center retrospective study, and case inclusion relies on past medical records and follow-up data, making it difficult to completely avoid information bias. Second, this study did not adopt random grouping, and whether patients entered the daytime surgery or inpatient pathway was affected by multiple factors such as comorbidities, anesthesia assessment results, family care ability and patients' willingness. Although there was no statistically significant difference in the comparison of baseline characteristics between the two groups, residual selection bias may still exist. Although the non-randomized design may introduce selection bias, the results of our multivariate regression analyses, which adjusted for baseline characteristics and potential confounders, consistently demonstrated the economic and clinical advantages of the day surgery model. This indicates that the observed benefits are robust even after accounting for residual confounding. Third, the sample size of this study is relatively limited, and the statistical power for low-incidence severe complications is insufficient, so the conclusion that “there is no difference in rare adverse events” still needs to be interpreted cautiously. Furthermore, based on the current sample size (*n* = 168), the statistical power may be insufficient to detect differences in rare complications such as cement leakage leading to neurological deficit or pulmonary embolism. Therefore, the conclusion of “no statistically significant differences in complications” should be interpreted cautiously, as it could represent a Type II error. Fourth, the follow-up time of this study mainly focuses on the medium and short term. Although it can reflect pain relief, functional recovery and some re-fracture cases, the evaluation of long-term spinal mechanical changes, long-term re-fracture risk and long-term quality of life improvement is still insufficient. Fifth, the statistics of direct medical costs are mainly based on in-hospital charging items, and indirect costs such as patients' transportation, lost work, family accompaniment and out-of-hospital rehabilitation have not been included, so the evaluation of the overall health economic value is still incomplete. Therefore, the conclusion is strictly limited to direct medical costs, and the overall economic advantage of the day surgery model may be underestimated. Sixth, patient satisfaction is assessed by a single scale, which may be affected by expectations, communication methods and subjective experience at discharge, resulting in certain measurement bias. Finally, this study is based on the management experience and standardized pathways of a single-center mature team, and the external generalizability of its results is still affected by hospital management level, anesthesia support ability, nursing resources and the perfection of the follow-up system. In the future, multicenter, prospective, preferably propensity score matching or randomized controlled trials should be carried out, combined with long-term quality of life and cost-effectiveness analysis, to further verify the optimal applicable scope of daytime surgery for OVCF percutaneous vertebroplasty.

In conclusion, day surgery is safe and feasible for percutaneous vertebroplasty in patients with osteoporotic vertebral compression fractures. For patients who are suitable for day management after standardized evaluation, their clinical efficacy is comparable to that of inpatient management, and it can significantly shorten hospitalization time, reduce direct medical costs, and improve patient satisfaction, showing good clinical application prospects.

## Data Availability

The original contributions presented in the study are included in the article/Supplementary Material, further inquiries can be directed to the corresponding author.

## References

[1] Huang H Yang M Fu Z Tan L. Efficacy and safety of curved vertebroplasty in osteoporotic vertebral compression fractures: a systematic review and meta-analysis. BMC Musculoskelet Disord. (2026) 27(1):390. 10.1186/s12891-026-09767-0 41896811 PMC13151077

[2] Creech-Organ DO J Organ DO B. Vertebral compression fractures. Am Fam Physician. (2026) 113(1):51–6. 41544281

[3] Jiang L Houston R Li C Siddiqi J Ma Q Wei S. Day surgery program at west China hospital: exploring the initial experience. Cureus. (2020) 12(7):e8961. 10.7759/cureus.8961 32766004 PMC7398727

[4] Clark W Bird P Gonski P Diamond TH Smerdely P McNeil HP. Safety and efficacy of vertebroplasty for acute painful osteoporotic fractures (VAPOUR): a multicentre, randomised, double-blind, placebo-controlled trial. Lancet. (2016) 388(10052):1408–16. 10.1016/S0140-6736(16)31341-1 27544377

[5] Nie B Wang Q Li B Ou N Yang Z. Exploration of percutaneous vertebroplasty in the treatment of osteoporotic vertebral compression fracture as day surgery: a retrospective study. Eur Spine J. (2021) 30(9):2718–25. 10.1007/s00586-021-06887-0 34075472

[6] An Z Dou J Mao W Wu B Zhang H Feng J. Percutaneous kyphoplasty for osteoporotic vertebral compression fractures performed in one-day surgery: safe and effective? Front Surg. (2025) 12:1636150. 10.3389/fsurg.2025.1636150 41141704 PMC12549701

[7] Luo J Xie C Fan D. Historical development and experience of day surgery in China: from the perspective of anesthesiologists. Paediatr Anaesth. (2025) 35(6):412–23. 10.1111/pan.15078 39921339 PMC12060084

[8] Sun N Chu Y Ge Z Liu Y. Percutaneous vertebral augmentation for osteoporotic vertebral compression fractures: minimally invasive techniques and clinical outcomes. Eur J Med Res. (2025) 30(1):1037. 10.1186/s40001-025-03311-x 41163108 PMC12570528

[9] Daher M Sebaaly A Sakr I Daniels AH Schoenfeld AJ. Diagnosis and management of osteoporotic vertebral compression fractures. J Bone Joint Surg Am. (2026) 108(5):345–54. 10.2106/JBJS.25.00201 41460925

[10] Stanak M Strohmaier C. Minimum volume standards in day surgery: a systematic review. BMC Health Serv Res. (2020) 20(1):886. 10.1186/s12913-020-05724-2 32948161 PMC7501608

[11] Haibier A Jie Y Yusufu A Shoukeer K Hang L Abudurexiti T. Effect of different cement distribution on the clinical efficacy of vertebral compression fractures in unilateral percutaneous vertebroplasty. Eur Spine J. (2025) 34(5):1673–84. 10.1007/s00586-024-08630-x 39945854

[12] Williams TD Adler T Smokoff L Kaur A Rodriguez B Prakash KJ. Bone cements used in vertebral augmentation: a state-of-the-art narrative review. J Pain Res. (2024) 17:1029–40. 10.2147/JPR.S437827 38505504 PMC10949389

[13] Hoffmann J Preston G Whaley J Khalil JG. Vertebral augmentation in spine surgery. J Am Acad Orthop Surg. (2023) 31(10):477–89. 10.5435/JAAOS-D-22-00958 36952673

[14] Rose LD Bateman G Ahmed A. Clinical significance of cement leakage in kyphoplasty and vertebroplasty: a systematic review. Eur Spine J. (2024) 33(4):1484–9. 10.1007/s00586-023-08026-3 37999769

[15] Wu Y Zhou Z Lu G Ye L Lao A Ouyang S. Risk factors for cement leakage after percutaneous vertebral augmentation for osteoporotic vertebral compression fractures: a meta-analysis. Int J Surg. (2025) 111(1):1231–43. 10.1097/JS9.0000000000001895 38978188 PMC11745741

[16] Lin YH Lin J Xu JY Lai BX He MH Zhu YR. What risk factors are associated with recurrent osteoporotic vertebral compression fractures after percutaneous vertebral augmentation? A meta-analysis. Clin Orthop Relat Res. (2025) 483(8):1528–39. 10.1097/CORR.0000000000003430 40036060 PMC12266891

[17] Sun D Wen Y Yu Q Long Y Liu Y Zhou Y. Prediction models for adjacent vertebral fractures after vertebral augmentation: a systematic review and meta-analysis. Eur Spine J. (2025) 34(5):1631–40. 10.1007/s00586-025-08785-1 40090978

